# Generation of transgenic rodent malaria parasites by transfection of cell culture-derived merozoites

**DOI:** 10.1186/s12936-017-1949-y

**Published:** 2017-08-01

**Authors:** Gesine Kaiser, Mariana De Niz, Paul-Christian Burda, Livia Niklaus, Rebecca Limenitakis Stanway, Volker Heussler

**Affiliations:** 1 Institute of Cell Biology, University of Bern, Bern, Switzerland; 2 Graduate School for Cellular and Biomedical Sciences, University of Bern, Bern, Switzerland; 3 Wellcome Centre for Molecular Parasitology, University of Glasgow, Glasgow, UK; 4 Bernhard Nocht Institute for Tropical Medicine, Hamburg, Germany

**Keywords:** *Plasmodium berghei*, Transfection, Liver stage-derived merozoites

## Abstract

**Background:**

Malaria research is greatly dependent on and has drastically advanced with the possibility of genetically modifying *Plasmodium* parasites. The commonly used transfection protocol by Janse and colleagues utilizes blood stage-derived *Plasmodium berghei* schizonts that have been purified from a blood culture by density gradient centrifugation. Naturally, this transfection protocol depends on the availability of suitably infected mice, constituting a time-based variable. In this study, the potential of transfecting liver stage-derived merozoites was explored. In cell culture, upon merozoite development, infected cells detach from the neighbouring cells and can be easily harvested from the cell culture supernatant. This protocol offers robust experimental timing and temporal flexibility.

**Methods:**

HeLa cells are infected with *P. berghei* sporozoites to obtain liver stage-derived merozoites, which are harvested from the cell culture supernatant and are transfected using the Amaxa Nucleofector^®^ electroporation technology.

**Results:**

Using this protocol, wild type *P. berghei* ANKA strain and marker-free PbmCherry_Hsp70_-expressing *P. berghei* parasites were successfully transfected with DNA constructs designed for integration via single- or double-crossover homologous recombination.

**Conclusion:**

An alternative protocol for *Plasmodium* transfection is hereby provided, which uses liver stage-derived *P. berghei* merozoites for transfection. This protocol has the potential to substantially reduce the number of mice used per transfection, as well as to increase the temporal flexibility and robustness of performing transfections, if mosquitoes are routinely present in the laboratory. Transfection of liver stage-derived *P. berghei* parasites should enable generation of transgenic parasites within 8–18 days.

**Electronic supplementary material:**

The online version of this article (doi:10.1186/s12936-017-1949-y) contains supplementary material, which is available to authorized users.

## Background

### Genetic manipulation of *Plasmodium* parasites

In the past 15 years, *Plasmodium* parasites have become greatly accessible for genetic manipulation [1–3], facilitated by the genome sequencing of human and rodent malaria parasites [4–6]. Advances continue and *Plasmodium knowlesi* parasites were recently successfully adapted for in vitro culture in human red blood cells including successful transfection, which resulted in efficiencies of up to 30% [7]. Transfection efficiencies of rodent *Plasmodium berghei* parasites have increased up to 1:1000 as a result of implementing the highly efficient non-viral Nucleofector^®^ technology [1, 8]. A major advantage of using the model organism *P. berghei* for *Plasmodium* research is the accessibility of the entire life cycle in vitro as well as in vivo, including the liver stage development. A further advantage is the availability of an almost complete genomic DNA library that originated from phage-based vectors, applicable for generation of knock-outs, and tagging of genes [9–11]. Methods for typical genetic manipulation, such as the generation of knock-outs and complemented parasites, fluorescent tagging of proteins and even conditional knock-outs, are available for both rodent and human *Plasmodium* parasites [9, 12–14]. Classically, transfection of DNA constructs into *P. berghei* parasites is performed into blood stage-derived schizonts and merozoites, and benefits from the fact that schizonts do not rupture in in vitro blood cultures and can thus be enriched and purified. Transfection of schizonts and free merozoites, compared to other asexual blood stages, is facilitated by the fact that DNA used for transfection has to cross only two or three membranes, namely the erythrocyte membrane (depending on whether or not merozoites have been released), the parasite plasma membrane (PM) and the nuclear membrane, instead of four, including the parasitophorous vacuole membrane [1]. The standard protocol for *P. berghei* transfection, requires the infection of two mice, which ideally should have a parasitaemia of about 3% usually achieved between day 5 and 7 after pre-infection. Once the parasitaemia has reached about 3%, blood stage parasites are taken into culture for 16–18 h and following this, schizonts are purified using a density gradient. Purified schizonts and merozoites are subsequently transfected using the Amaxa Nucleofector^®^ electroporation technology [1, 8]. This study took advantage of the fact that the merozoite stage of *Plasmodium* parasites is not restricted to the blood stage, but is also produced at the end of liver stage development. The *Plasmodium* liver stage is characterized by an immense expansion of the parasite population. Intriguingly, a single sporozoite that has infected a host hepatocyte can mature into thousands of progeny merozoites [15]. At the end of exo-erythrocytic parasite development, merozoites are released from the parasitophorous vacuole (PV) into the hepatocyte cytoplasm. This leads to the detachment of the infected host cell from its neighbouring cells and in in vitro cultures, to detachment of the infected cells, which then float freely in the culture supernatant. Merosomes, sacs containing infectious merozoites, are subsequently extruded from the detached cell and are also found in the cell culture supernatant [16, 17]. Single detached cells of in vitro-cultured *P. berghei* parasites were recently described to harbour an average of about 4500 individual merozoites [16, 18]. In a previous study by Stanway et al. individual merosomes or detached cells were collected and used for sub-cloning of transgenic parasites, thereby greatly contributing to the reduction of animals used to achieve clonal transgenic parasite lines [17]. This work presents an established and optimized protocol for transfection of liver stage-derived schizonts and merozoites, which equally aims to reduce the number of animals used for the generation of transgenic parasite lines.

## Methods

### Animal work statement

Experiments were conducted in strict accordance with the guidelines of the Swiss Tierschutzgesetz (TSchG; Animal Rights Laws) and approved by the ethical committee of the University of Bern (Permit Number: BE109/13). Balb/c mice used in experiments were between 6 and 10 weeks of age and were either bred in the central animal facility of the University of Bern, or were supplied from Harlan Laboratories or Charles River.

### Culturing of HeLa cells

HeLa cells (a gift from Robert Menard, Pasteur Institute, Paris) were grown in MEM (minimum essential medium) with Earle’s salts (Bioconcept, cat. no 1-31F01-I), supplemented with 10% heat inactivated foetal calf serum—FCS (Bioswisstec, Cat. No. S4150), 1% penicillin/streptomycin (Bioconcept, Cat. No. 4-01F00-H) and 1% l-glutamine (Bioconcept, 5-10K00-H) in a humid incubator with 5% CO_2_ at 37 °C. Using accutase (Sigma-Aldrich, Cat. No. A6964), cells were passaged twice a week.

### Infection of mosquitoes with *Plasmodium berghei*

A *P. berghei* blood stabilate was injected intraperitoneally into a naïve Balb/c mouse (referred to as ‘pre-infection mouse’). When this mouse had reached a parasitaemia of 1–4%, 50 µl of infected blood, diluted with 150 µl PBS, were injected intravenously into a mouse (referred to as ‘feed mouse’) that 3 days prior to that had been intraperitoneally injected with 200 µl phenylhydrazine (6 mg ml^−1^ in PBS) (Sigma-Aldrich, Cat. No. 114715). When the feed mouse, had reached a parasitaemia of at least 7%, usually 3–4 days after infection, with most of the circulating parasites being gametocytes, the mouse was anaesthetized and used to allow about 100–150 female *Anopheles stephensi* mosquitoes to feed for 1 h. Infected mosquitoes were kept at 20.5 °C and 80% humidity and fed with 8% fructose containing 0.2% PABA (Sigma-Aldrich, Cat. No. 100536). Infected mosquitoes were dissected to obtain sporozoites for the detached cell transfection protocol between day 16 and 24 post blood feed.

### In vitro infection of HeLa cells with *Plasmodium berghei*

Infection of HeLa cells with sporozoites required that mosquitoes infected with either *P. berghei* (ANKA strain) wild type parasites or any other selection-marker-free *P. berghei* parasite line were available.

On the day of the infection, the parasites in these mosquitoes were between day 16 to 24 post-blood feed. For one transfection (if more transfections were intended cells were seeded accordingly), HeLa cells were seeded into three wells of a 24-well plate (Greiner Bio-one, Cat. No. 662160) with 2 × 10^4^ cells per well and cultured in complete MEM culture medium. On the following day at about 16.00, 3–5 infected mosquitoes per transfection were anesthetized with chloroform vapour. Anesthetized mosquitoes were briefly dipped into 70% (vol/vol) ethanol and then transferred to PBS.

For removal of salivary glands, mosquitoes were successively put into 200 µl of infection medium [complete MEM culture medium containing 2.5 μg ml^−1^ amphotericin B (Sigma-Aldrich, Cat. No. A2942)] on a slide and the head was carefully removed using surgical forceps. The two sets of each three salivary gland lobes were isolated from the head or mosquito body, and transferred into an Eppendorf tube containing 20 µl of infection medium, kept on ice until infection of HeLa cells. Salivary glands were mechanically disrupted using a pestle (Sigma-Aldrich, Cat. No. Z359947) driven by a Cordless motor (Sigma-Aldrich, Cat. No. Z359971), using approximately 10 pulses of 1–2 s. Next, 300 µl of infection medium were added and the number of sporozoites was estimated using a Neubauer chamber.

Per anticipated transfection, 50,000 sporozoites were used, contained in a volume of 600 µl infection medium. After removing the medium from the three wells to be infected, 200 µl of sporozoite-containing infection medium were added to each of the three wells. To allow fast settling of the sporozoites, the 24-well plate was spun for 1 min at 200*g*. Following 2 h of incubation time (at 37 °C with 5% CO_2_), infected cells were washed with 500 µl pre-warmed infection medium to remove mosquito debris and sporozoites that had not been infected. Cells were then incubated with 500 µl infection medium in a humid incubator at 37 °C with 5% CO_2_. To avoid fungal and bacterial growth, the infection medium was exchanged at 24 and 48 h post infection (hpi).

### Harvesting detached cells and merosomes

At about 62–65 hpi (about 09.00 at day 3 post-infection) it was verified by bright-field microscopy that detached cells/merosomes were present in the cell culture supernatant. Detached cells and merosomes are characterized by their round shape. They contain thousands of small merozoites, which, in viable merosomes and detached cells, are constantly moving. If only few detached cells had been formed, infection duration was extended to up to 67 hpi. To harvest detached cells and merosomes, the supernatant of each well was gently pipetted up and down three times and finally the medium of the three infected wells was pooled into one Eppendorf tube. Parasites were pelleted at 16,000*g* for 10 s, the direction of the tube was changed by 180° and the tube was spun again for 10 s at 16,000*g*. Successful centrifugation was verified by the presence of a small semi-transparent/white pellet visible at the bottom of the tube. If more than one transfection was planned, all sets of detached cells were prepared before the transfection was performed.

### Transfection of liver stage-derived merozoites

The transfection procedure was adapted from the previously published protocol by Janse et al. [8]. Mice to be injected with transfected parasites were pre-warmed using an infrared lamp for about 5–10 min prior to the injection, to allow the tail vein to dilate. 5–10 µg of DNA (contained in a maximum volume of 10 µl) were added to 100 µl Nucleofector solution 88A6 (Lonza, Cat. No. VVPA-1002 KT) and the same was done for each of the constructs to be transfected. Next, using a 1000-µl pipette, the supernatant was carefully removed from one detached cell-containing Eppendorf tube, without touching the detached cell pellet. Subsequently, 100 µl of DNA-containing Nucleofector solution 88A6 were added and the detached cells and merosomes were cautiously resuspended. Resuspended parasites were transferred from the Eppendorf tube into an electroporation cuvette and placed into the Amaxa Nucleofector device. Parasites were transfected using the program U33 of the Amaxa Nucleofector device. Immediately after transfection, 50 µl of RPMI1640 culture medium (Bioconcept, Cat. No. 1-41F01-I) were added to the transfected parasites. Following this, the whole volume of the cuvette (about 150 µl) was transferred into an Eppendorf tube using the provided Pasteur plastic pipette. Using an insulin syringe, the entire suspension was injected into the tail vein of a naïve Balb/c mouse (6–10 weeks of age) and the previous steps were repeated until all constructs were transfected.

### Drug selection and collection of transgenic parasites

One day after transfection, drugs suitable for selection of transgenic parasites were given to infected mice (depending on the transfected construct), as previously published [8]. Drug selection was continued until transgenic parasites had reached a parasitaemia of 2% or higher. From 5 days after the transfection onwards, the parasitaemia of mice injected with transfected parasites was checked by thin blood smear. If the transfected construct encoded fluorescent or luminescent markers expressed under a promoter active in the blood stage, fluorescence or luminescence was also checked.

### Detection of parasites by fluorescence

To stain the parasite DNA, a drop of blood from the infected mouse was added to 50 µl of PBS containing 1 μg ml^−1^ Hoechst 33342 (Sigma-Aldrich, Cat. No. B2261) in PBS. Parasites were analysed under a fluorescence microscope using the appropriate filters and an increasing magnification of between 200× and 1000×.

### Detection of parasites by luminescence (NanoLuc)

Parasite detection by luminescence was performed as described before [18]. Briefly, a drop of blood from the infected mouse was added to 20 µl of PLB (1 × Passive Lysis Buffer; Promega, Cat. No. E1941) contained in one well of a black, flat-bottom, 96-well plate (Greiner Bio-One, Cat. No. 655900). As a negative control, blood was collected from a non-infected mouse. A 1:50 dilution of the NanoGlo substrate was prepared in NanoGlo^TM^ luciferase assay buffer (Promega, Cat. No. N1120), 50 µl of the dilution were added to the sample and the luminescence signal was read within 5 min.

### Genotyping of transfected parasites

Genomic DNA of transfected parasites was purified. Integration PCR was performed according to the schematic in Fig. 6. DNA sequences of primers used are shown in Table 1.

**Table 1:** Primers used for genotyping in Fig. 6

Primer	Sequence (5′–3′)
1	GTGTAGTAACATCAGTTATTGTGTG
2	ATACTGTATAACAGGTAAGCTGTTATTGTG
3	TTTCCCAGTCACGACGTTG
4	CTTAGTGTTTTGTATTAATGTCGATT TG
QCR2	AGGGGCAAATACCAAAGTTGTGT
QCR1	ACGCATATTCACGAGTTTCACA
GW2	CTTTGGTGACAGATACTAC

## Results

**Fig. 1:**
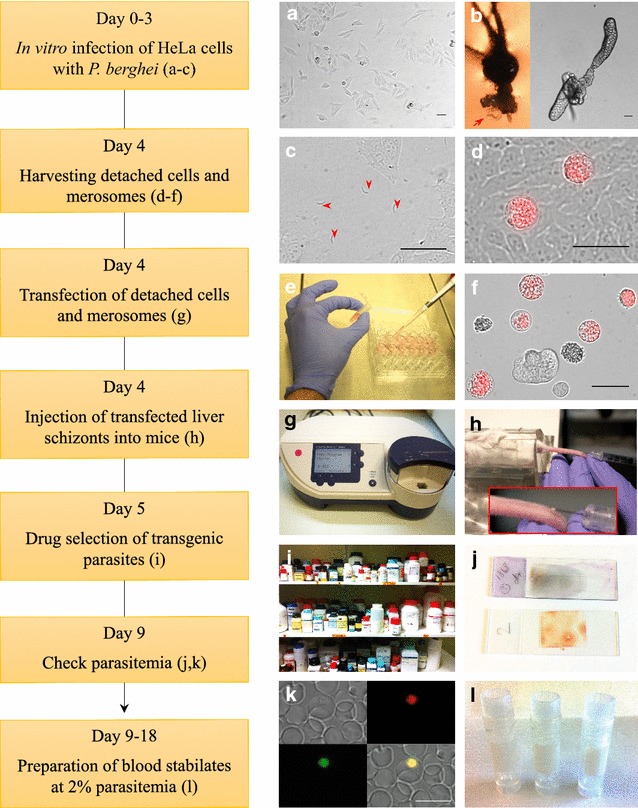
Workflow and visualization of critical protocol steps. **a** Optimal density of seeded HeLa cells 24 h post seeding. **b** Representative images of dissection of mosquito salivary glands (*red arrow*) attached to the head (*left*) or removed (*right*). **c** HeLa cells infected with *P. berghei* sporozoites at a density representing sporozoite numbers used in the here described protocol. **d** HeLa cells infected with PbmCherry_Hsp70_ 65 hpi, prior to detached cell (*red*) collection. **e** Collection of detached cells at 65 hpi. **f** Representative image of collected PbmCherry_Hsp70_ detached cells (*red*) at 65 hpi and uninfected HeLa cell debris (unlabelled). **g** Amaxa^®^ Nucleofector^®^ II device with transfection program U33. **h** Intravenous injection of transfected parasites into a naïve mouse. **i** Drug selection of transgenic parasites. **j** Checking the parasitaemia by Wright stain or fluorescence. **k** Monitor transgenic parasites by fluorescence. **l** Preparation of blood stabilates. *Scale bars* in all images are 50 µm

**Fig. 2:**
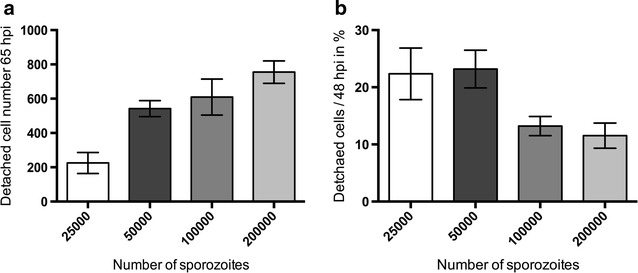
Experimental optimization of sporozoite numbers for HeLa cell infection to obtain maximal numbers of detached cells/merosomes. **a**, **b** HeLa cells were infected with different numbers of PbmCherry_Hsp70_ parasites and counted at 48 hpi. Detached cells and merosomes were counted in the supernatant at 65 hpi. **a** Total number of detached cells formed at 65 hpi depending on the number of sporozoites used for infection. **b** Percentage of detached cells formed in relation to parasite count at 48 hpi was calculated. Shown are means with errors depicted as 95% confidence intervals, n = 3 in biological triplicates

This work presents an established protocol that allows efficient transfection of liver stage-derived merozoites in so-called detached cells and merosomes (Fig. 1). This protocol is based on the fact that in vitro, *P. berghei*-infected cells detach from their neighbouring cells and float into the cell culture supernatant. Thus, they can be easily purified by harvesting and then centrifuging the supernatant. After initially succeeding in transfecting detached cells/merosomes, the next aim was to optimize the formation and yield of detached cells to quantify the corresponding number of merozoites used, and to standardize the protocol in a user-friendly way. As a first step, the optimal number of sporozoites to be used for host cell infection to maximize the generation of detached cells was determined (Fig. 2). As expected, with a constant number of host cells, the number of detached cells increases as the number of sporozoites used per infection increases. This increase is not linear and reaches a plateau with about 50,000 sporozoites. On average, using 50,000 sporozoites, about 550 detached cells were obtained when harvesting at 65 hpi (Fig. 2a**)**, which is about 20% of the parasites that were present at 48 hpi (Fig. 2b). When higher sporozoite numbers were used, the number of detached cells formed also increased but the conversion rate of parasites present at 48 hpi to detached cells dropped considerably (Fig. 2b), which suggests sub-optimal parasite development.

**Fig. 3:**
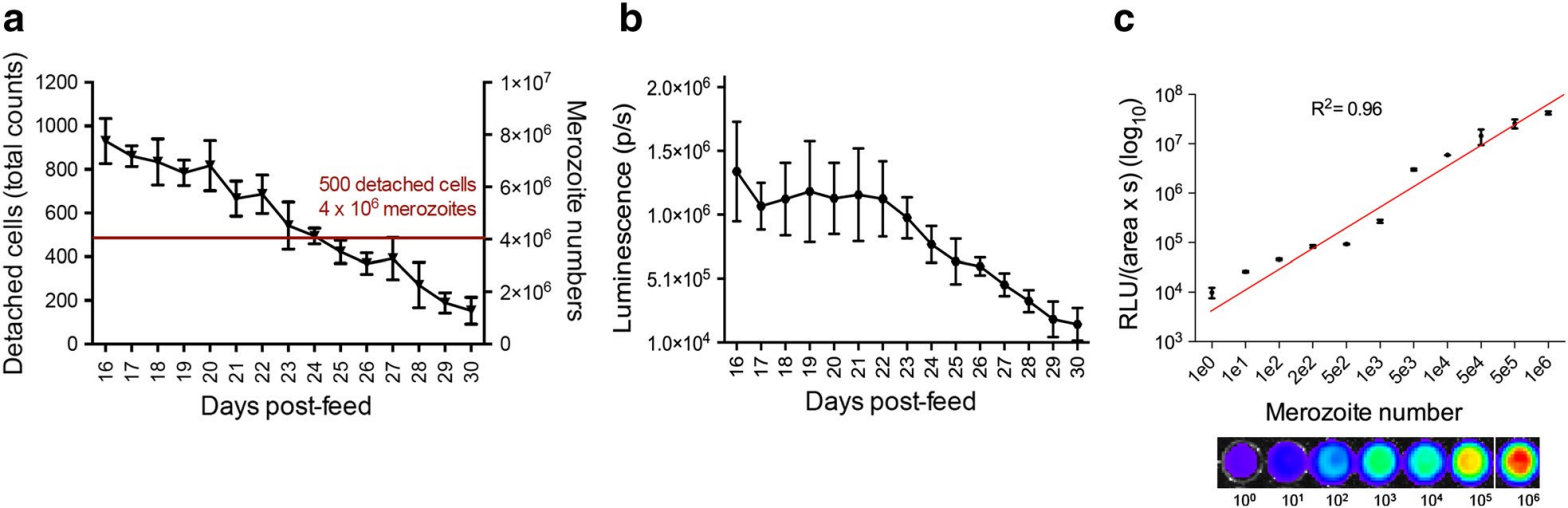
Microscopic and luminescence-based determination of detached cell and merozoite number, dependent on the parasite age in infected mosquitoes. **a** The relation of the parasite age in infected mosquitoes to the number of detached cells obtained and the relative merozoite number was investigated from day 16 to day 30. A critical cut off (*red line*), in detached cell numbers obtained is at 500 detached cells (4 × 10^6^ merozoites), as the success rate of detached cell transfections using fewer than 500 detached cells decreases. Seeding and infections were performed according to the present protocol, collected detached cells were counted using a fluorescent microscope at 65 hpi and merozoite number was determined using a luminescence based assay, n = 3. *Error bars* denote SD. **b** Determination of luminescence of detached cells obtained over time, using sporozoites from day 16 to day 30 post-blood feed. Detached cells of PbmCherry_Hsp70_-NLuc parasites were obtained and collected according to the present protocol. Aside from quantification of detached cell numbers by fluorescence, luminescence was used to estimate the number of detached cells used for transfection from day 16 onwards, following mosquito feeds. Luminescence correlates with number of detached cells contained, and suggests that at days 16–23, the number of viable merozoites remains relatively constant, while a significant decrease in luminescence is identified from day 24 onwards (denoting less detached cells and less merozoites). Values shown are the result of three independent experiments repeated in triplicate. *Error bars* denote SD. **c** PbmCherry_Hsp70_-NLuc detached cells were collected and disrupted to release merozoites. The total number of merozoites of a single detached cell was counted, and a limiting dilution was performed to achieve one merozoite, which was lysed and its luminescence measured. The radiance calculated was 9.9 × 10^4^ p s^−1^ cm^−2^. Performing a limiting dilution from 1 to 10^6^ merozoites we defined that a positive, significant (R^2^ = 0.96) correlation exists between merozoite number and the log_10_ of the relative light units of luminescence. This in turn allowed us to estimate the total number of merozoites in single individual detached cells and of the complete population of detached cells used for transfection. Values shown are the result of ten separate limiting dilutions from three independent experiments

Having established the optimal sporozoite number to use for infection, the effect of age of the parasite in the mosquito on the number of detached cells/merosomes formed and the corresponding merozoite number was determined (Fig. 3a). Detached cell formation was quantified, using sporozoites obtained from mosquitoes from day 16 to day 30 post-blood feed. Based on previous experiments, it was found that the success rate and efficiency of detached cell transfection starts to drop considerably when using fewer than 500 detached cells/merosomes (4 × 10^6^ merozoites). The minimum was set to 500 detached cells/merosomes and beyond day 24 post-blood feed, numbers of detached cells formed dropped below this critical level. Accordingly, harvesting sporozoites from mosquitoes between day 16 and day 24 post-blood feed results in the sufficient formation of detached cells. Thus, sporozoites can be used over a period of nine consecutive days, during which transfections can be flexibly and robustly planned and performed, as the processes from seeding of cells to liver stage-derived merozoite transfection takes place over a period of only 4 days.

For detached cell quantification, in addition to a fluorescence microscopy-based assay, a luminescence-based assay was adapted to determine the numbers of detached cells and merozoites (Fig. 3). Using this assay, it was calculated that about 4.5–8.5 × 10^6^ merozoites are used per transfection (Fig. 3a).

**Fig. 4:**
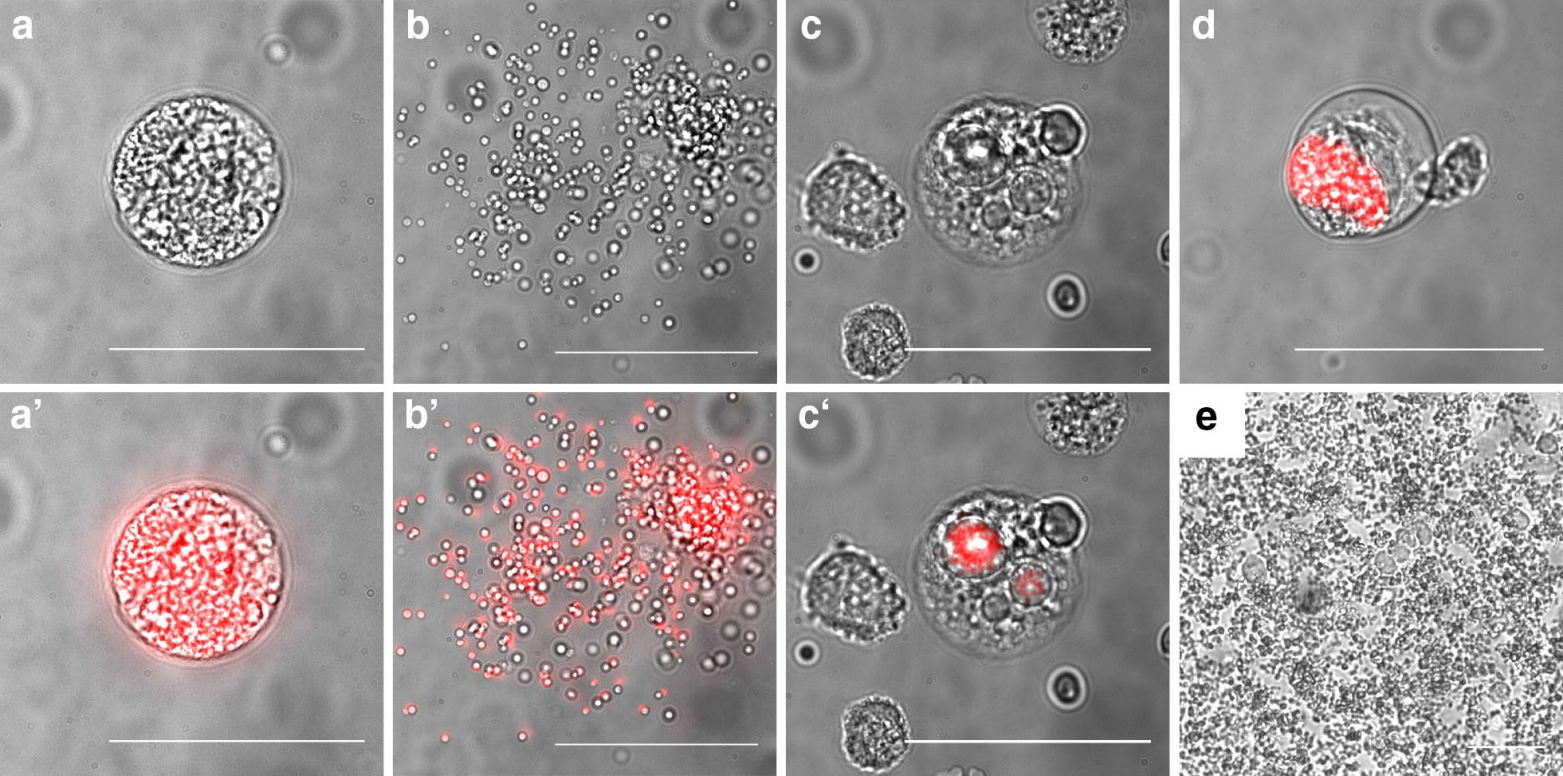
Representative microscopic images of detached cells. **a** Bright field and **a′** fluorescence merged image of a normal detached cell, **b** and **b′** of a ruptured detached cell and **c**, **d** examples of detached cells with abnormal morphology, **c**, **c′** of merofusosomes [25] and **d** of absent merozoite formation. **e** Example of a fungal contamination of *Plasmodium* infected cells. *Scale bars* are 50 µm

However, experiments also showed that not only the quantity but also the viability and quality of detached cells and contained merozoites is key to successful transfection. Microscopic assessment is the fastest and most efficient way to assess detached cell morphology (Fig. 4). Transfection of detached cells had a better efficiency when most of the detached cells contained viable and motile merozoites (Fig. 4a, b; Additional file 1: Movie S1) and only few abnormal detached cells were present (Fig. 4c, d). Furthermore, it is also important to microscopically confirm that cells have not been contaminated during the process of infection with sporozoites, which can introduce bacteria and fungi, contained in the mosquito debris, to the cell culture (Fig. 3e). If contamination has occurred, transfection should not be performed, as contaminated detached cells must not be injected into mice.

**Fig. 5:**
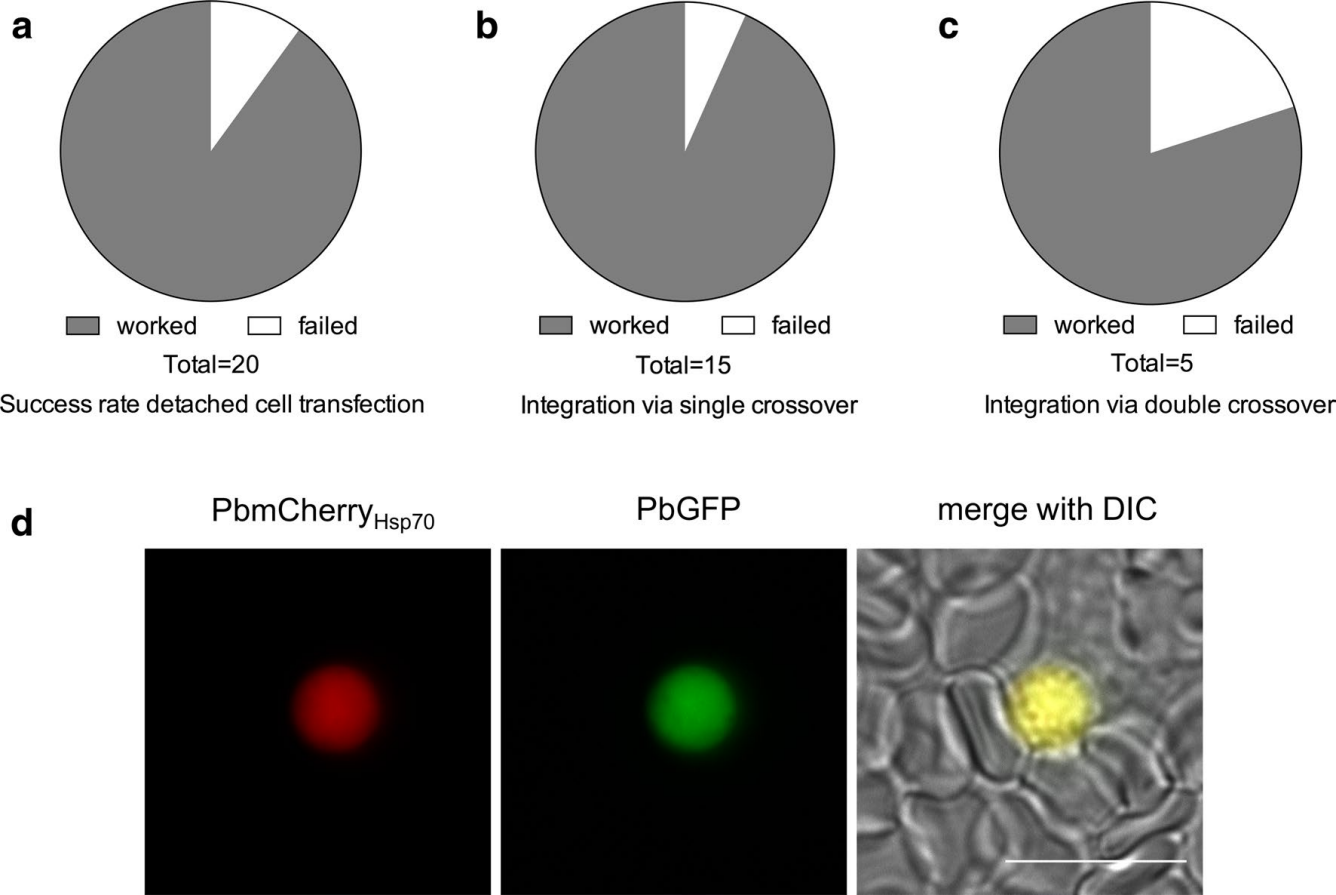
Success rate of detached cell transfection of *Plasmodium berghei* parasites. Detached cells and merosomes of wild type or PbmCherry_Hsp70_ parasites were transfected according to the present protocol. **a** Illustration of the total success rate of detached cell transfection independent of parasites used and constructs transfected. **b** Success rate of detached cell transfection using DNA constructs allowing integration via either single or **c** double crossover homologous recombination. **d** Example of successfully transfected PbmCherry_Hsp70_ parasites with a construct allowing expression of cytoplasmic GFP. *Scale bar* is 10 µm

**Fig. 6:**
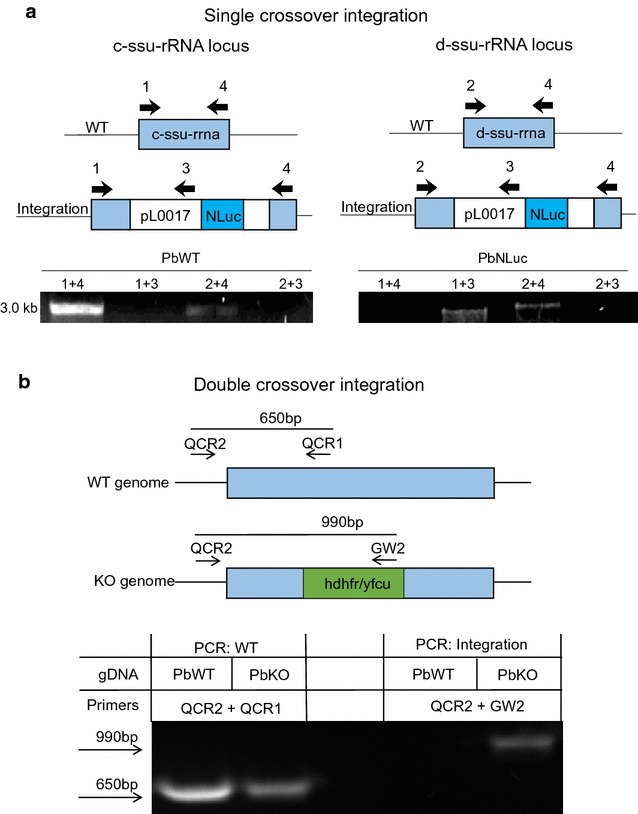
Integration PCR of parasite lines generated by detached cell transfection using **a** single or **b** double crossover integration. **a** *P. berghei* parasites were transfected with pL0017-NLuc [18], a construct that allows integration by single crossover into the *P. berghei c*-*ssu*-*rRNA* or *d*-*ssu*-*rRNA* locus. PCR amplification was used to verify integration of the plasmid into the *P. berghei* genome. The schematic shows the possible annealing regions of the primer pairs for the different parasite genotypes that could result. If the construct did not integrate into the parasite genome the primer pairs 1 + 4 for the *c*-*ssu*-*rRNA* or 2 + 4 for the *d*-*ssu*-*rRNA* locus generated a 3 kb PCR product. If the construct was successfully integrated, the primer pairs are too far apart (>14 kb) to generate a PCR product. Primer pairs 1 + 3 and 2 + 3 are designed to confirm integration, a PCR product of 3 kb is generated only if the construct has integrated. PCR results of the PbNLuc parasites show integration into the *c*-*ssu*-*rRNA* locus, as the band for primer pair 1 + 4 is absent and the primer pair 1 + 3 gives a 3 kb band, there is no integration into the *d*-*ssu*-*rRNA* locus, which shows the same PCR products as for the WT parasites. **b** *P. berghei* parasites were transfected with PbGEM-336027, the construct facilitates integration of the hdhfr/yfcu cassette via double crossover into the PBANKA_1145300 gene locus, resulting in a knock-out (KO). The primer pair QCR2 + QCR1 results in a 650 bp fragment, if parasites have a PBANKA_1145300 WT genotype. The primer pair QCR2 + GW2 is designed to show integration. A 990 bp PCR product is generated only, if the construct has integrated at the correct site of the parasite genome. The PCR results show integration of the hdhfr/yfcu cassette into the PBANKA_1145300 gene locus. Transfected parasites are present in a mixed population of parasites having a PBANKA_1145300 KO genotype and PBANKA_1145300 WT genotype

This protocol was successfully used for transfection of DNA constructs facilitating integration into the parasite genome by single crossover homologous recombination (Figs. 5, 6; Table 2) to introduce DNA constructs for cytoplasmic GFP expression and expression of a luciferase reporter gene in addition to a previously introduced cytoplasmic mCherry expression cassette [18]. Moreover, DNA constructs facilitating integration into the parasite genome by double crossover homologous recombination, were also successfully transfected, for example for the generation of knock-out and complemented parasites (Figs. 5, 6; Table 2). DNA constructs were successfully transfected into different parasite backgrounds, such as wild type parasites, selection-marker-free cytoplasmic mCherry expressing parasites [20] and knock-out parasites. In conclusion, all techniques used to transfect blood stage parasites can also be used to manipulate liver stage-derived merozoites.

**Table 2:** shows the individual detached cell transfections performed that are summarized in Fig. 5a–c with information about the transfected construct, the parasite line that was transfected, the type of integration, the success rate, how often the transfection was performed and at which day after the transfection the mice became positive

Construct	Parasite line	Integration	Success rate	Number of transfections	Day mice became positive
PbSBP1KO (P0B90-GFP) [19]	mCherry_Hsp70_ [20]	Double crossover	50% (2)	2	7
PbSBP1KO (P0B90-GFP) [19]	mCherry_Hsp70_FLuc_ef1a_ [21]	Double crossover	100% (1)	2	6
PbSBP1KO (P0B90-GFP) [19]	WT	Double crossover	100% (1)	1	7
PfSBP1 (PL0017-mCherry) [19]	PbSBP1KO [19]	Single crossover	100% (1)	1	8
PbGEM-336027	mCherry_Hsp70_ [20]	Double crossover	100% (1)	1	21
PbNLuc (PL0017) [18]	WT	Single crossover	100% (1)	1	5
PbNLuc (PL0017) [18]	mCherry_Hsp70_ [20]	Single crossover	100% (2)	2	4
PbNLuc (PL0017) [18]	mCherry_Hsp70_FLuc_ef1a_ [21]	Single crossover	66.6% (3)	3	4–5
PL0017 GFP [22]	mCherry_Hsp70_ [20]	Single crossover	100% (8)	8	5–12

## Discussion

The protocol described here allows the generation of transgenic *P. berghei* parasites. If infected mosquitoes are routinely accessible, the protocol offers a flexible experimental procedure and the potential to reduce the number of mice used per transfection. In addition to transfection of *P. berghei* blood stages, the new protocol provides an optimized usage of *P. berghei* parasites for transfection. Using liver stage-derived merozoites for transfection, mouse numbers used should be reduced most efficiently when the protocol is used for high numbers of transfections. Transfection of liver stage-derived schizonts and merozoites of either wild type *P. berghei* ANKA or transgenic selection-marker-free *P. berghei* ANKA parasites has been proven successful for transfection of DNA constructs facilitating integration into the parasite genome by both single- and double-crossover homologous recombination. Transfections performed with DNA constructs for integration by single homologous recombination had a slightly higher success rate than transfections performed with DNA constructs that integrated by double crossover homologous recombination. Most likely this is related to the mechanism of integration and whether or not expression can be episomal, rather than being a consequence of the transfection procedure itself [23, 24].

Transfection of liver stage-derived detached cells and merosomes should be successful for all genetic modifications that can be achieved by single or double crossover homologous recombination, such as expression of proteins fused with fluorescent, luminescent or other markers and interference with protein function by knock-out, conditional knock-out, modification, over-expression or replacement. In addition, this protocol should also be successful when introducing secondary genetic modifications such as complementation of previously knocked-out genes, additional marker fused proteins and double knock-outs.

A major advantage of the protocol is that liver stage-derived merozoites can be collected and transfected in less than 20 min, which is time saving and presumably beneficial for parasite viability. This is mainly because mature detached cells/merosomes can be directly collected from the cell culture supernatant and need no further purification by density gradient centrifugation as is needed for the purification of blood stage schizonts.

The standard protocol for blood stage transfection published by Janse et al. recommends using a minimum of 10^6^ schizonts, corresponding to about 8–18 × 10^6^ single merozoites per transfection [8]. Using the here-described protocol for transfection of liver stage-derived merozoites, about 4.5–8.5 × 10^6^ merozoites are used per transfection, depending on the number of detached cells obtained. An important observation was that efficient parasite development leading to the formation of detached cells is highly dependent on the age of parasites in infected mosquitoes. Therefore, it is recommended that mosquitoes are used between days 16 and 24 after blood feeding. Being able to use infected mosquitoes for 9 consecutive days to obtain detached cells, parasite transfections can be performed more flexibly in terms of timing and can be planned more robustly than is possible when performing blood stage transfections, which greatly depend on the parasitaemia of infected mice to be used for blood stage cultures. The liver stage development in vitro is independent of host circadian rhythms and accordingly, infections to obtain liver stage derived merozoites can be performed at any time of the day, offering flexible transfection conditions.

To obtain sporozoites needed for liver stage cultivation, a total of two mice is required: a pre-infection mouse in which infection is initially established from a blood stage stabilate of the parent *P. berghei* line, and a phenylhydrazine-treated recipient mouse (referred to as ‘feed mouse’), which will be infected with the donor’s blood, and thereafter used to feed the mosquitoes. Since only low blood volumes are passaged from the pre-infection mouse into the feed mouse, several feed mice can be infected from a single pre-infection mouse, to increase the number of mosquitoes that can be infected and thus the number of sporozoites that can be generated. In fact, in laboratories that routinely perform mouse infections with *P. berghei*, an additional pre-infection mouse is not required further reducing the number of experimental animals needed for the transfection.

During the optimization process of the protocol it was established that about 50,000 sporozoites are needed to obtain sufficient numbers of liver stage-derived merozoites per transfection. On average this sporozoite number can be obtained from three infected mosquitoes [18]. Normally, about 100–120 mosquitoes are fed on a single feed mouse. With a routine infection rate of about 70–80%, about 80 infected mosquitoes were obtained. Per transfection, three mosquitoes that on average harbour about 50,000 sporozoites are dissected and used for the infection of HeLa cells. This infection results in about 500–1000 detached cells, which is sufficient for one transfection. Accordingly, it is possible to perform about 27 transfections with the number of infected mosquitoes that result from sacrificing a single feed mouse. Conversely, with the standard protocol used for transfection of blood stage-derived schizonts [8], which in theory requires 1 mouse per 4–5 transfections, performance of the same amount of transfections would require five mice.

Therefore, the protocol presented here is highly beneficial when intending to perform transfections in large scale. Transfection of liver stage-derived merozoites and schizonts has the potential to reduce the mouse usage by about fivefold in laboratories that have routine access to infected mosquitoes compared to the current protocol based on blood stage transfection. Since the in vitro liver stage development of *Plasmodium* takes place in cell culture plates, it is very suitable for scaling up the experimental design. Potential critical points could be that sporozoites, following isolation, should be used sufficiently quickly to ensure efficient host cell infection. Later in the experimental procedure, the time between detached cell purification and transfection is critical for parasite survival. Therefore, it is proposed that host cell infection with sporozoites and transfection of liver stage-derived parasites should be performed in sets, with a maximum of five transfections per set. Nevertheless, multiple infections could be prepared at time intervals if many transfections are planned.

## Conclusions

So far, only one stage of *Plasmodium* berghei parasites, blood stage schizonts, could be transfected. Here a transfection protocol is described using liver stage-derived merozoites of *P. berghei*, which can be generated in vitro and in contrast to transfection of blood stage schizonts do not require sophisticated purification steps. The protocol described here provides more flexibility in terms of when and how many transfections can be performed. Moreover, transfection of liver stage-derived merozoites has the potential to result in a considerable reduction of mice used, if used by a lab that routinely has access to infected mosquitoes.

## Additional file

**Additional file 1: Movie S1.** Motile merozoites within detached cells. Detached cells containing marker-free PbmCherry_Hsp70_ parasites (red) were harvested at 65 hpi and analysed using a fluorescence microscope. Images were acquired every 5 s. Scale bar is 10 μm.

## Abbreviations

DNA: deoxyribonucleic acid
GFP: green-fluorescent protein
hpi: hours post infection
PABA: para-aminobenzoic acid
PM: plasma membrane
PV: parasitophorous vacuole
PBS: phosphate-buffered saline
